# Dysregulation of Astrocytic HMGB1 Signaling in Amyotrophic Lateral Sclerosis

**DOI:** 10.3389/fnins.2018.00622

**Published:** 2018-08-29

**Authors:** Liliana Brambilla, Francesca Martorana, Giulia Guidotti, Daniela Rossi

**Affiliations:** Laboratory for Research on Neurodegenerative Disorders, IRCCS Istituti Clinici Scientifici Maugeri (ICS Maugeri), Pavia, Italy

**Keywords:** astrocytes, HMGB1, ALS, motor neuron, GDNF, BDNF, neuroinflammation

## Abstract

Astrocytes have emerged as critical elements for the maintenance and function of the central nervous system. The expression on their cell membrane of RAGE and TLR4 receptors makes astrocytes susceptible to High-mobility group box 1 (HMGB1), a nuclear protein typically released in the extracellular milieu by living cells experiencing physiological stress conditions or by damaged cells. Here, we show that the interaction of HMGB1 with normal spinal cord astrocytes induces the astrocytic production of neurotrophic factors, particularly brain-derived neurotrophic factor (BDNF) and glial cell line-derived neurotrophic factor (GDNF). Multiple investigations suggest a role for HMGB1 in amyotrophic lateral sclerosis (ALS). Yet, no mechanistic information on the implication of HMGB1 signaling in this disorder is currently available. We demonstrate that non-transgenic and transgenic SOD1^WT^ spinal motor neurons exhibit only a basal nucleus-to-cytoplasm shuttling of the HMGB1 protein. Conversely, in SOD1^G93A^ ALS mouse spinal cords, HMGB1 significantly translocates from the nucleus to the cytoplasm of motor neurons, thereby suggesting that it may be eventually released in the extracellular environment during the progression of the disease. We postulate that extracellular HMGB1 can paracrinally interact with the neighboring astrocytes in an attempt to counteract the neurodegenerative process. Yet, at variance with normal cells, SOD1^G93A^-expressing astrocytes show impaired capacity to raise BDNF and GDNF levels upon HMGB1 stimulation. Our data suggest that HMGB1 have a potential to promote neuroprotective actions by healthy astrocytes. However, this neurotrophic response is disrupted in ALS astrocytes. This indicates that diseased astroglial cells may exacerbate motor neuron degeneration in ALS because of the loss of their neurosupportive functions.

## Introduction

Astrocytes are the most abundant glial cell population in the central nervous system (CNS) and are essential to maintain CNS homeostasis and function. Owing to the expression of a large repertoire of pattern recognition receptors (PRRs), astrocytes can sense multiple endogenous damage-associated molecular patterns (DAMPs) (Bsibsi et al., 2002; Farina et al., 2005; Park et al., 2006; Gorina et al., 2011; reviewed in Rossi, 2015), including the High-mobility group box 1 (HMGB1). HMGB1 is a nuclear protein that can be actively secreted by living cells experiencing physiological stress conditions or passively released by dying cells. Once in the extracellular milieu, HMGB1 can signal into the microenvironment through the interaction with various PRRs, such as receptor for Advanced Glycation End-Products (RAGE) and Toll-like Receptor 2 (TLR2) and 4 (TLR4) (Vénéreau et al., 2015; Rider et al., 2017). Three conserved redox-sensitive cysteines within the amino acid sequence determine the interaction of HMGB1 with these receptors, thereby influencing the extracellular activities of this protein (Harris et al., 2012; Antoine et al., 2014). Hence, reduced cysteines render HMGB1 chemoattractant, whereas the formation of an intramolecular disulfide bond enables the partially oxidized HMGB1 isoform, named “disulfide-HMGB1,” a pro-inflammatory cytokine-stimulating mediator (e.g., increasing the production of Tumor Necrosis Factor α, TNFα) (Venereau et al., 2012; Yang et al., 2012; reviewed in Vénéreau et al., 2015). Consistent with this latter function, disulfide-HMGB1 has been described to sustain neuroinflammation and related neurodamaging events in various CNS disorders (Fang et al., 2012). In the injured CNS, astrocytes are key regulators of the neuroinflammatory reaction. In addition to release a plethora of pro-inflammatory mediators, they can secrete a variety of trophic factors. These include brain-derived neurotrophic factor (BDNF) and glial cell line-derived neurotrophic factor (GDNF) (Schaar et al., 1993; Appel et al., 1997; Dougherty et al., 2000; Ikeda et al., 2001; Chen et al., 2006; reviewed in Rossi, 2015; Colombo and Farina, 2016). BDNF and GDNF have been long described as major regulators of survival, maintenance and regeneration of specific neuronal populations in the adult brain, so as to be considered valuable therapeutic options for various neurodegenerative diseases, including amyotrophic lateral sclerosis (ALS) (Allen et al., 2013). In the present study, we hypothesized that disulfide-HMGB1, released within the CNS after physiological stressor events, may act as a paracrine/autocrine mediator on the astrocytes and drive protective actions on the neighboring neurons via the release of trophic factors (Choi et al., 2017). We found that normal astrocytes, but not astroglial cells from a mouse model of ALS, respond to HMGB1 stimulation by enhancing their production of BDNF and GDNF.

## Materials and Methods

### Transgenic Mice

Transgenic mice expressing the human SOD1^WT^ [B6SJL-Tg(SOD1)2Gur/J – 002298] or SOD1^G93A^ [B6SJL-TgN(SOD1-G93A)1Gur/J – 002726] (Gurney et al., 1994) were purchased from The Jackson Laboratories. The colonies were maintained by breeding hemizygote SOD1^WT^ or SOD1^G93A^ males to wild-type C57Bl6/SJL hybrid females (Charles River Laboratories).

Genomic DNA was extracted from mouse tail biopsies and used as DNA template in genotyping PCR analysis. Offspring positive for the SOD1^WT^ or SOD1^G93A^ transgene were identified using primers 5′-SOD1 (5′-CATCAGCCCTAATCCATCTGA) and 3′-SOD1 (3′-CGCGACTAACAATCAAAGTGA). All animal studies were carried out in accordance with the recommendations of the European Council Directive 2010/63/EU and the Italian Animal Welfare Act for the use and care of laboratory animals and were approved by the Animal Welfare Committee of the University of Pavia.

### Primary Astrocyte Cultures and Pharmacological Treatments

Primary astrocyte cultures (>99% GFAP-positive) were prepared from the spinal cord of newborn mice (non-Tg littermates, SOD1^WT^ and SOD1^G93A^), as previously described (Rossi et al., 2008; Martorana et al., 2012). Cells were maintained in Minimal Essential Medium (MEM, Gibco, Life Technologies) supplemented with 10% fetal bovine serum (FBS, Gibco, Life Technologies), 2% L-Glutamine, 2% Glucose, 1% Penicillin/Streptomycin, 1% Antibiotic antimycotic solution (Euroclone). Once the cells reached the confluence, they were re-plated at the optimal density (2 × 10^4^ cells/well) in 24-well plates. After 1 week, cells were pre-incubated for 1 h in the presence or in the absence of the RAGE inhibitor FPS-ZM1 (50 nM, Calbiochem) or the TLR4 antagonist CLI-095 (5 μM, InvivoGen). Then, astrocytic cultures were stimulated with or without recombinant HGMB1 (3 μg/ml, R&D Systems) for 6 h and scraped either for RNA extraction or for BDNF and GDNF ELISAs.

### Real-Time RT-qPCR

Total RNA was extracted from primary astrocytic cultures using ReliaPrep^TM^ RNA Cell Miniprep System (Promega) according to manufacturer’s guidelines. One microgram of total RNA was reverse-transcribed using iScript cDNA Synthesis Kit (Bio-Rad Laboratories) according to manufacturer’s guidelines. The resulting cDNAs (2–10 ng) were analyzed by quantitative PCR using the SsoFast EvaGreen Supermix on a CFX96 Real-Time PCR Detection System (Bio-Rad Laboratories). The following primers were used: GDNF (forward: 5′ CCGCTGAAGACCACTCCCT; reverse: 5′ TAATCTTCAGGCATATTGGAGTCACT), BDNF (forward: 5′ CAAACAAGACACATTACCTTCCTGC; reverse: 5′ CTTCTCACCTGGTGGAACATTG), RAGE (forward: 5′ ACTACCGAGTCCGAGTCTACC; reverse: 5′ GTAGCTTCCCTCAGACACACA), TLR4 (forward: 5′ GGACTCTGATCATGGCACTG; reverse: 5′ CTGATCCATGCATTGGTAGGT) and hypoxanthine guanine phosphoribosyl transferase (HPRT) (forward: 5′ TGAATCACGTTTGTGTCATTA; reverse: 5′ TTCAACTTGCGCTCATCTTAG), as housekeeping gene. Relative expression was determined by the 2^-ΔΔct^ method and normalized to HPRT gene expression.

### BDNF and GDNF ELISAs

BDNF and GDNF ELISAs for intracellular protein quantification were performed according to manufacturer’s guidelines (BDNF and GDNF E_max_^®^ ImmunoAssay System, Promega). Briefly, cells were homogenized in lysis buffer [137 mM NaCl, 20 mM TRIS pH 8.0, 1% NP40, 10% glycerol, 100 nM Na_3_VO_4_, supplemented with Protease Inhibitor Cocktail Tables Complete (Roche)] and centrifuged for 5 min at 10,000 ×*g* at 4°C. Supernatants were acidified and neutralized to pH 7.6 and then processed in 96-well plates, as follows.

Plates were coated with anti-BDNF or anti-GDNF monoclonal antibodies, blocked and incubated with BDNF or GDNF standards, respectively, or samples. Then, plates were sequentially incubated with anti-human BDNF or GDNF polyclonal antibodies and an anti-IgY-peroxidase conjugate, followed by the tetramethylbenzidine solution for color development. Reactions were stopped with 1N HCl and the absorbance at 450 nm was measured with an iMark plate reader (Bio-Rad Laboratories). Levels of BDNF or GDNF in cell lysates were normalized to total protein and expressed as picograms per mg of total protein (pg/mg of protein). The assays sensitivity ranged from 8 to 500 pg/ml for BDNF and from 16 to 1000 pg/ml for GDNF.

### Tissue Processing and Immunohistochemical Analysis

Mouse spines from non-Tg, SOD1^WT^ and SOD1^G93A^ mice were taken and immersed in 4% buffered paraformaldehyde for 24 h. Spinal cords were extracted and the lumbar tract was removed, paraffin embedded and sectioned at 10 μm. On selected sections, the following immunohistochemical stainings were carried out: non-phosphorylated neurofilament H (SMI32, mouse monoclonal antibody, 1:500, Covance) and HMGB1 (rabbit monoclonal antibody, 1:500, Abcam). Nuclei were labeled with Hoechst 33342 (Sigma-Aldrich). For quantitative analysis, lumbar spinal cord images were captured using a 40× objective on a DM5000 B microscope (Leica Microsystem) equipped with a digital camera DFC 310 FX (Leica Microsystem). The number of nuclei positively stained for HMGB1 in motor neurons was determined on images from lumbar spinal cord sections double stained for HMGB1 and SMI32. The results were expressed as percentage of the total number of cells taken into consideration.

### Statistical Analysis

Data are represented as mean ± SEM and statistical significance was verified using GraphPad Prism^®^ software. Statistical significance was assessed by *t*-test for comparisons between two groups; one-way or two-way ANOVA followed by Bonferroni *post hoc* test were used for comparisons of multiple groups.

## Results

**FIGURE 1:**
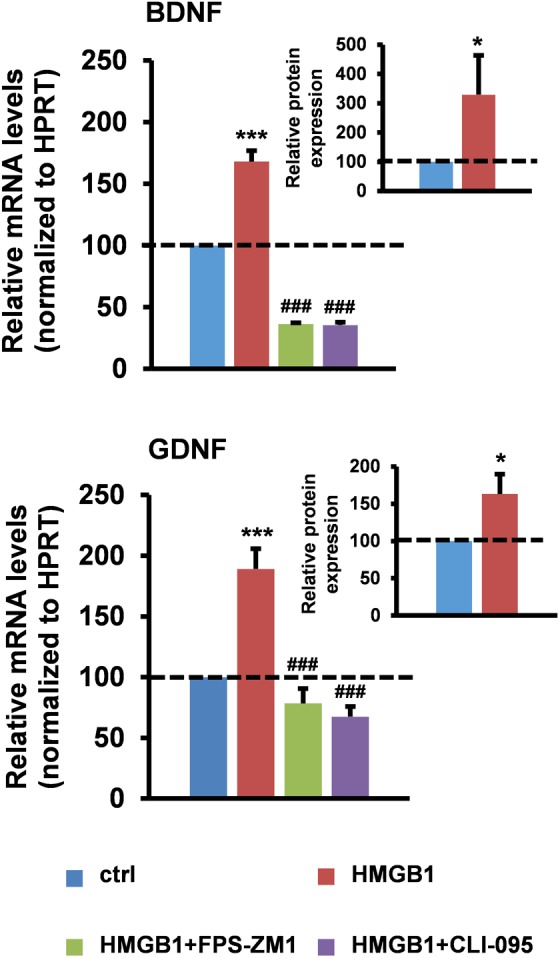
High-mobility group box 1 (HMGB1) stimulates trophic factor expression in mouse spinal cord astrocytes. Astrocytes from the spinal cord of wild-type mice were pre-incubated for 1 h in the absence or in the presence of the RAGE antagonist FPS-ZM1 (50 nM) or the TLR4 antagonist CLI-095 (5 μM), and then treated with recombinant disulfide-HMGB1 (3 μg/ml) for 6 h. Total RNA was extracted and analyzed by RT-qPCR. Values (mean ± SEM) were normalized relative to hypoxanthine guanine phosphoribosyl transferase (HPRT) and expressed as percentage of untreated cells (ctrl) (main charts). ^∗∗∗^*p* < 0.0001 HMGB1 vs. crtl; ^###^*p* < 0.0001 HMGB1+FPS-ZM1 or +CLI-095 vs. HMGB1, one-way ANOVA followed by Bonferroni *post hoc* test, *n* = 3–17 experiments, in triplicate. Intracellular BDNF and GDNF protein levels from wild-type spinal cord astrocytes were quantified by ELISAs after treatment with recombinant disulfide-HMGB1 (3 μg/ml) for 6 h. Values (mean ± SEM) were expressed as percentage of untreated cells (ctrl) (inset charts). ^∗^*p* < 0.05 HMGB1 vs. ctrl, two-tailed unpaired *t*-test, *n* = 6 experiments, in duplicate.

To directly test the hypothesis that healthy astrocytes can respond to HMGB1 stimulation by increasing their neurotrophic properties, we first treated primary astrocytes from wild-type mouse spinal cord with disulfide-HMGB1 (3 μg/ml, 6 h) and we analyzed the astrocytic expression of BDNF and GDNF at the messenger RNA (mRNA) and protein level. We found that stimulation with disulfide-HMGB1 induced a significant increase in the astrocytic levels of the transcripts coding for the two trophic factors when compared to untreated cells, as assessed by reverse transcription quantitative polymerase chain reaction (RT-qPCR) (**Figure 1**). In parallel experiments, we evaluated by ELISA, on the same type of astrocytic preparations, the intracellular levels of the two trophic factors upon treatment with disulfide-HMGB1 (3 μg/ml, 6 h). As shown in **Figure 1**, we confirmed a concomitant and significant boost in the intracellular levels of the BDNF and GDNF proteins upon treatment. This suggests that astroglia can respond to stimulation with partially oxidized HMGB1 by increasing their trophic support. Because disulfide-HMGB1 can typically promote the production of TNFα, we next ensured that the protein isoform used in our experiments was actually disulfide-HMGB1 by analyzing the increase in the production of this pro-inflammatory cytokine. We found that the expression of TNFα mRNA was 2.3-fold higher in the HMGB1-treated cells as compared to untreated astrocytes (**Supplementary Figure S1**). Next, we investigated the PRR(s) involved in this process. Since TLR2 was undetectable in ALS astrocytes (Casula et al., 2011), we specifically focused on TLR4 and RAGE. Cells were pre-treated (1 h) in the absence or in the presence of the RAGE inhibitor FPS-FM1 (50 nM) or the TLR4 antagonist CLI-095 (5 μM), and then exposed to disulfide-HMGB1 (3 μg/ml, 6 h). RT-qPCR analysis revealed that RAGE and TLR4 are both implicated in the trophic factor expression, as indicated by the fact that both receptor antagonists significantly suppressed the HMGB1-dependent increment in BDNF and GDNF mRNA levels (**Figure 1**). The two receptor inhibitors exerted similar inhibitory effects also toward the expression of TNFα (**Supplementary Figure S1**).

**FIGURE 2:**
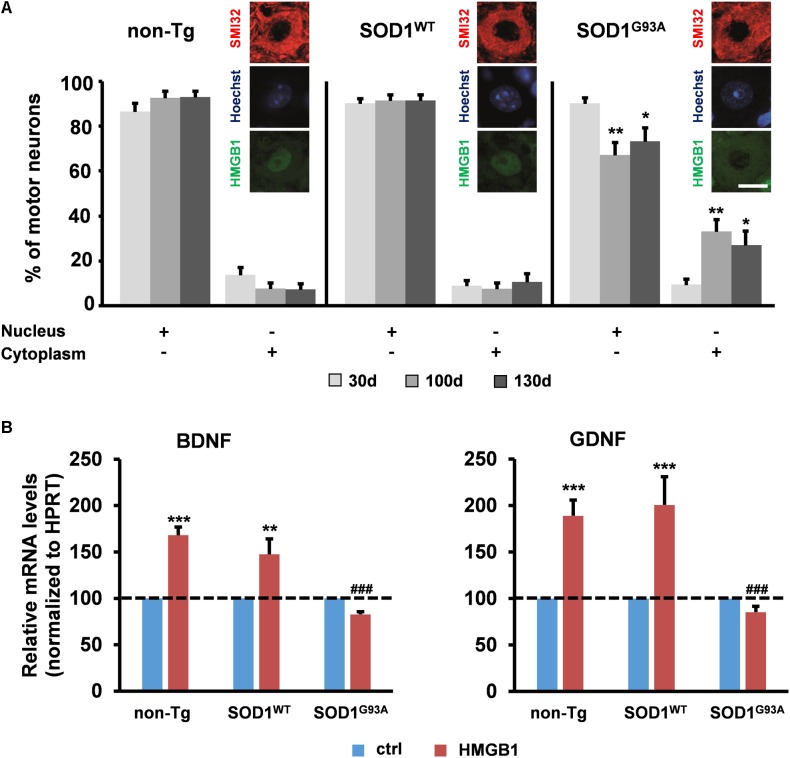
Potential implication of HMGB1 signaling in motor neuron-astrocyte communication in ALS. **(A)** The number of motor neurons showing HMGB1 into the nucleus or the cytoplasm was determined by immunohistochemistry on spinal cord sections from non-transgenic (non-Tg) or SOD1^WT^ and SOD1^G93A^ transgenic mice taken at 30 days (30 days, asymptomatic stage), ∼100 days (100 days, onset of motor deficits), and 130 days of age (130 days, symptomatic stage). Data (mean ± SEM) are expressed as percentage of motor neurons with nuclear or cytoplasmic HMGB1 on the total number of analyzed cells. ^∗∗^*p* < 0.01 and ^∗^*p* < 0.05 vs. 30 days SOD1^G93A^; two-way ANOVA, followed by Bonferroni *post hoc* test, *n* = 3 mice per age, *n* = 190–313 motor neurons per genotype and age. Insets are representative immunofluorescent images of spinal cord sections double immunostained for SMI32 (red) and HMGB1 (green); nuclei were stained with Hoechst 33342 (blue). Scale bar, 10 μm. **(B)** Expression of BDNF and GDNF was determined in primary astrocytes from the spinal cord of non-Tg, SOD1^WT^ and SOD1^G93A^ mice. Cells were exposed to disulfide-HMGB1 (3 μg/ml) for 6 h. Total RNA was extracted and analyzed by RT-qPCR. Values (mean ± SEM) were normalized relative to HPRT and expressed as percentage of control (ctrl), i.e., the corresponding culture type challenged with saline. ^∗∗∗^*p* < 0.001, ^∗∗^*p* < 0.01 vs. ctrl; ^###^*p* < 0.001 vs. HMGB1 in non-Tg and SOD1^WT^ astrocytes; two-way ANOVA, followed by Bonferroni *post hoc* test, *n* = 5–17 experiments, in triplicate.

Various studies in patients and animal models suggest the implication of HMGB1 in ALS, a disease characterized by upper and lower motor neuron degeneration (Casula et al., 2011; Juranek et al., 2015). Yet, no mechanistic information is currently available on the role of the HMGB1 signaling in this disorder. Several lines of evidence indicate that motor neuron degeneration in ALS is non-cell-autonomous and requires the interaction with the neighboring astrocytes (Clement et al., 2003; Lepore et al., 2008; Yamanaka et al., 2008; Papadeas et al., 2011; Wang et al., 2011; Kondo et al., 2014). To investigate the potential involvement of HMGB1 in motor neuron-astrocyte communication, we evaluated the subcellular distribution of this protein in spinal motor neurons from non-transgenic mice (non-Tg) or transgenic mice over-expressing either the wild-type superoxide dismutase 1 (SOD1^WT^) or the ALS-linked mutant SOD1^G93A^ protein at the most critical stages of disease progression, i.e., 30 days (asymptomatic stage), ∼100 days (onset of motor deficits) and ∼130 days of age (symptomatic stage). We found that the number of motor neurons with nuclear HMGB1 remained constant over time in non-Tg and SOD1^WT^ spinal cords, with only a basal translocation of the protein in the cytoplasm (**Figure 2A**). At variance with this, we identified a progressive decrease in the amount of motor cells showing immunopositive nuclei for HMGB1 and a concomitant increase in the number of motor neurons exhibiting cytoplasmic HMGB1 in spinal cords from SOD1^G93A^ mice (**Figure 2A**). Because HMGB1 is normally located in the nucleus and translocates to the cytoplasm and extracellular space after traumatic events (Maroso et al., 2010; Choy et al., 2014; Sun et al., 2014; Agalave and Svensson, 2015), our data suggest that HMGB1 may be eventually released from stressed/damaged motor neurons into the extracellular milieu during ALS progression.

To determine whether extracellular HMGB1 can act on neighboring ALS astroglia, we exposed primary spinal cord astrocytes from non-Tg, SOD1^WT^ or SOD1^G93A^ mice to exogenous disulfide-HMGB1 (3 μg/ml, 6 h), so as to mimic paracrine actions of the potentially secreted protein. The expression levels of BDNF and GDNF transcripts were subsequently analyzed. We found that non-Tg and SOD1^WT^ astrocytes responded with an increased expression of both trophic factors, whereas SOD1^G93A^ astrocytes were unable to react to HMGB1 stimulation in terms of capacity to up-regulate the expression of BDNF and GDNF (**Figure 2B**). Next, we investigated whether these results could be ascribed to an impairment in RAGE and TLR4 signaling. Primary spinal cord astrocytes of the three aforementioned genotypes were thus treated with disulfide-HMGB1 (3 μg/ml, 6 h). The expression levels of the two receptors transcripts were then analyzed by RT-qPCR. We found that only non-Tg and SOD1^WT^ astrocytes exhibited an up-regulation of the two receptors, whereas ALS astrocytes responded with a trend toward a decrease of RAGE and TLR4 expression (**Supplementary Figure S2**). These results indicate that the protective signaling pathway triggered by extracellular disulfide-HMGB1 in the astrocytes is impaired in ALS.

## Discussion

Several lines of evidence suggest that the nuclear protein HMGB1 can be tonically secreted by living cells undergoing physiopathological stress (reviewed in Vénéreau et al., 2015). Once in the extracellular space, HMGB1 can act as a paracrine and/or autocrine mediator to counteract the stressor events, thereby contributing to restore a physiological microenvironment around the suffering cells and orchestrating cellular recovery. In the present study, we investigated the activities of partially oxidized disulfide-HMGB1 in the context of the CNS. We found that this protein isoform is able to play a key role as a pro-survival molecule. In particular, we showed that exogenous disulfide-HMGB1 can increase the production of the neurotrophic factors BDNF and GDNF by healthy spinal cord astrocytes, an event that is strictly dependent on TLR/RAGE signaling. HMGB1 can normally signal through diverse receptors, including TLR2, TLR4, and RAGE. Since TLR2 is undetectable in ALS astrocytes (Casula et al., 2011), we focused on TLR4 and RAGE, confirming a role for these two receptors in promoting the HMGB1-dependent neurotrophic activity. These observations are fully consistent with studies by others, describing the capacity of HMGB1 to promote gene expression programs that include the up-regulation of trophic factors, for example in the case of tumor angiogenesis (van Beijnum et al., 2013).

Various observations suggest that HMGB1 initiates a positive feedback loop that amplifies the neuroinflammatory and neurodegenerative processes in human and rodent ALS (Casula et al., 2011). Yet, no information is available about the specific HMGB1-dependent pathway(s) involved in this disease. To address this issue, here we focused on the SOD1^G93A^ ALS mouse model. We found that, in ALS motor cells, HMGB1 translocates from the nucleus into the cytoplasm over time. Because cytoplasmic accumulation generally precedes HMGB1 secretion, our finding indicates that HMGB1 may be eventually released from stressed/damaged motor neurons during ALS progression, potentially engaging a paracrine action onto neighboring astroglia. Conversely, non-Tg and SOD1^WT^ motor neurons exhibit only a basal nucleus-to-cytoplasm shuttling of the protein. Recently, we reported that ALS astrocytes do not lose their intrinsic ability to produce neurotrophic factors, as shown by the fact that stimulation with the pro-inflammatory cytokine TNFα boosted GDNF expression in a similar way to wild-type strains (Brambilla et al., 2016). Thus, here we investigated whether extracellular disulfide-HMGB1 likewise maintained its competence to prompt the expression of BNDF and GDNF in ALS astroglia. At variance with TNFα, we found that the SOD1^G93A^ cells were unable to respond to disulfide-HMGB1 stimulation with an increased production of both trophic factors. This event may be caused by functional deficits in the astrocytic TLR4 and RAGE signaling pathways, possibly determined by the presence of mutant SOD1. We postulate that the impairment of these signaling cascades makes ALS astrocytes unable to carry out their neurosupportive functions in response to disulfide-HMGB1 stimulation, and this may exacerbate motor neuron degeneration.

These observations are in line with previous evidence suggesting that astrocytes can contribute to neurodegeneration and disease progression in ALS (Yamanaka et al., 2008; Papadeas et al., 2011; Wang et al., 2011). More specifically, they support the concept that the functional imbalance of astroglial cells cannot be considered as a marginal event in ALS, but should be attentively evaluated in view of the intimate anatomical and functional relationships of these cells with motor neurons (Rossi et al., 2008; Rossi, 2015). According to the present literature, the reaction of the astrocytes to ALS is twofold: on the one hand, they can be directly toxic to motor neurons via the release of neurodamaging molecules (Di Giorgio et al., 2007, 2008; Nagai et al., 2007; Marchetto et al., 2008); on the other hand, astrocytes can worsen motor neuron degeneration indirectly by losing their homeostatic and neurosupportive functions (Rothstein et al., 1992, 1995; Van Damme et al., 2007; Cassina et al., 2008; Martorana et al., 2012). An example of the latter case is the widely described defect in the expression of the astrocyte-specific glutamate transporter EAAT2/GLT-1, which is crucial for controlling the physiological uptake of extracellular glutamate in the CNS (Rothstein et al., 1992, 1995; Bruijn et al., 1997; Howland et al., 2002). This may prolong the activity of the neurotransmitter at the synaptic cleft, thereby sensitizing both motor neurons and astrocytes to physiological levels of glutamate and causing their mutual degeneration in ALS (Rossi et al., 2008; Martorana et al., 2012). This phenomenon can be aggravated by additional functional deficits of the astrocytes, including those affecting their ability to produce trophic factors, which compromise the microenvironment of these two cell populations. Altogether, this amount of evidence confirms a role for the astrocytes in driving motor neuron degeneration in ALS and strongly suggest that they should be considered major cellular targets for therapeutic intervention.

## Author Contributions

DR conceived the study. LB, FM, and GG performed the experiments. LB, FM, and DR analyzed the results and wrote the manuscript. All authors read and approved the final manuscript.

## Conflict of Interest Statement

The authors declare that the research was conducted in the absence of any commercial or financial relationships that could be construed as a potential conflict of interest.
