# Supplementary material for: Dysregulation of Astrocytic HMGB1 Signaling in Amyotrophic Lateral Sclerosis
Source: Front Neurosci. 2018 Aug 29;12:622. doi: 10.3389/fnins.2018.00622 (PMC6123379; doi:10.3389/fnins.2018.00622)
Supplement: Supplementary file 1 [file Data_Sheet_1.PDF]

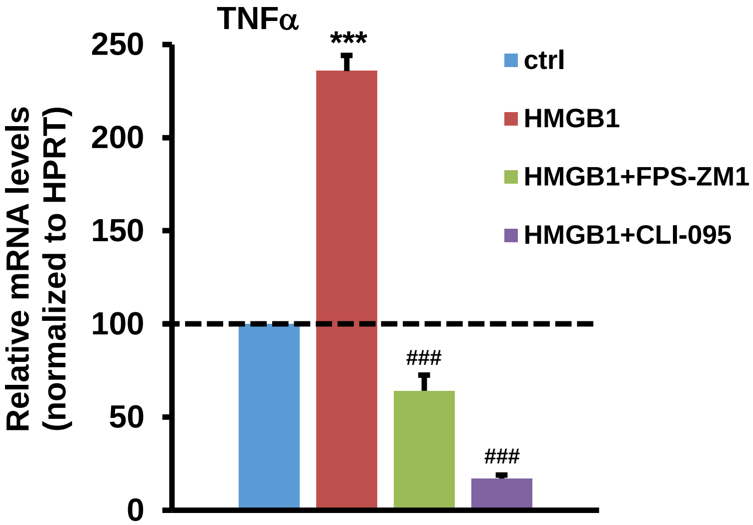

Supplementary Figure 1: Disulfide-HMGB1 induces TNFα expression in mouse spinal cord astrocytes. Expression of TNFα was determined in primary astrocytes from the spinal cord of wild-type mice after stimulation with recombinant disulfide-HMGB1. Cells were pre-incubated for 1h with or without the RAGE antagonist FPS-ZM1 (50 nM) or the TLR4 antagonist CLI-095 (5 µM), and then treated with recombinant disulfide-HMGB1 (3 µg/ml) for 6h. Total RNA was extracted and analyzed by RT-qPCR. Values (mean ± s.e.m.) were normalized relative to hypoxanthine guanine phosphoribosyl transferase (HPRT) and expressed as percentage of untreated cells (ctrl), i.e. the corresponding culture type challenged with saline. $^{***}p<0.0001$ HMGB1 *vs.* ctrl; $^{\#\#\#}p<0.0001$ HMGB1+FPS-ZM1 or +CLI-095 *vs.* HMGB1, one-way ANOVA followed by Bonferroni post-hoc test, n = 3-17 experiments, in triplicate.

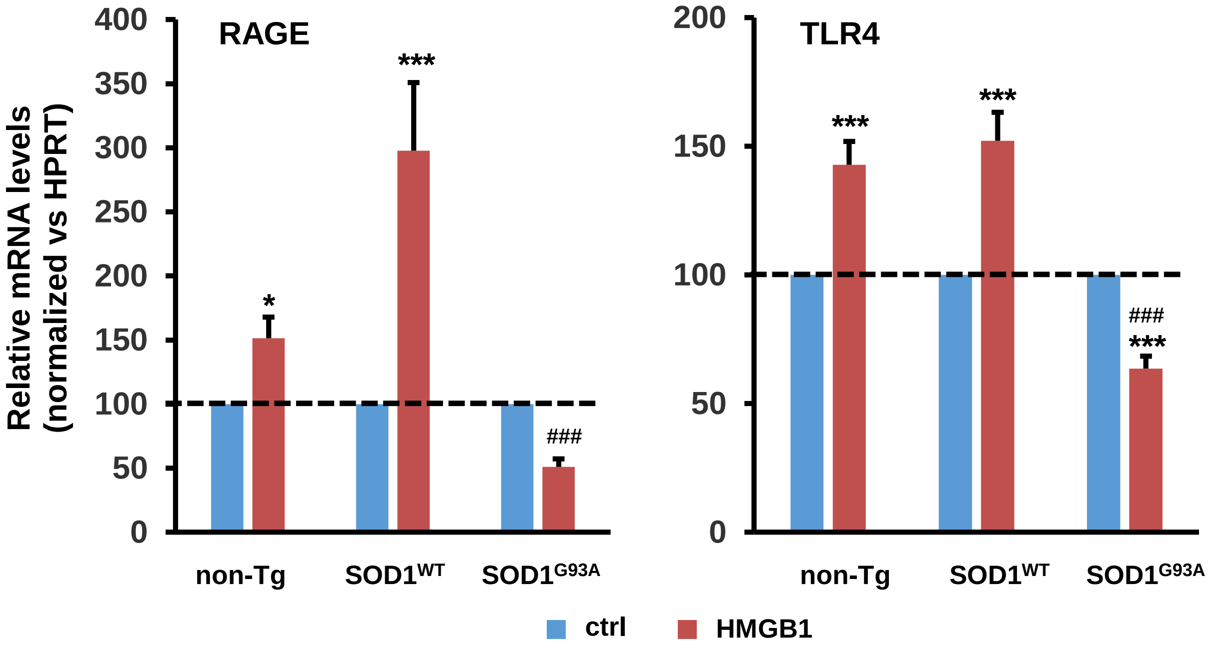

Supplementary Figure 2. Disulfide-HMGB1 is unable to induce the expression of RAGE and TLR4 in ALS mouse spinal cord astrocytes. Expression of the two receptors was determined in primary astrocytes from the spinal cord of non-Tg, $SOD1^{WT}$ and $SOD1^{G93A}$ mice after stimulation with recombinant disulfide-HMGB1 (3 µg/ml) for 6h. Total RNA was extracted and analyzed by RT-qPCR. Values (mean ± s.e.m.) were normalized relative to HPRT and expressed as percentage of untreated cells (ctrl), i.e. the corresponding culture type challenged with saline. *p<0.05 and ****p*<0.001 HMGB1 *vs.* ctrl; $^{###}p<0.001$ HMGB1 in $SOD1^{G93A}$ *vs.* HMGB1 in non-Tg and $SOD1^{WT}$ astrocytes; two-way ANOVA followed by Bonferroni post-hoc test, n = 3-10 experiments, in triplicate.
